# A large-scale microelectromechanical-systems-based silicon photonics LiDAR

**DOI:** 10.1038/s41586-022-04415-8

**Published:** 2022-03-09

**Authors:** Xiaosheng Zhang, Kyungmok Kwon, Johannes Henriksson, Jianheng Luo, Ming C. Wu

**Affiliations:** Department of Electrical Engineering and Computer Sciences, University of California, Berkeley, Berkeley, CA USA

**Keywords:** Optical sensors, Silicon photonics, Imaging and sensing

## Abstract

Three-dimensional (3D) imaging sensors allow machines to perceive, map and interact with the surrounding world^1^. The size of light detection and ranging (LiDAR) devices is often limited by mechanical scanners. Focal plane array-based 3D sensors are promising candidates for solid-state LiDARs because they allow electronic scanning without mechanical moving parts. However, their resolutions have been limited to 512 pixels or smaller^2^. In this paper, we report on a 16,384-pixel LiDAR with a wide field of view (FoV, 70° × 70°), a fine addressing resolution (0.6° × 0.6°), a narrow beam divergence (0.050° × 0.049°) and a random-access beam addressing with sub-MHz operation speed. The 128 × 128-element focal plane switch array (FPSA) of grating antennas and microelectromechanical systems (MEMS)-actuated optical switches are monolithically integrated on a 10 × 11-mm^2^ silicon photonic chip, where a 128 × 96 subarray is wire bonded and tested in experiments. 3D imaging with a distance resolution of 1.7 cm is achieved with frequency-modulated continuous-wave (FMCW) ranging in monostatic configuration. The FPSA can be mass-produced in complementary metal–oxide–semiconductor (CMOS) foundries, which will allow ubiquitous 3D sensors for use in autonomous cars, drones, robots and smartphones.

## Abstract (web summary)

A large focal plane switch array is constructed to steer the laser beam of a LiDAR system, leading to 3D imaging with 16,384 pixels, improving the resolution and coverage of solid-state LiDARs.

## Main

Autonomous systems powered by artificial intelligence will have a transformative impact on our society. 3D sensors that directly measure the coordinates, shapes and velocities of objects have seen an increasing number of applications in autonomous vehicles, drones and robots^3,4^. LiDAR systems can work in darkness and offer high resolution and high accuracy thanks to the short wavelength of light^5^. Small-form-factor LiDARs with single-photon avalanche diode arrays^6–8^ have started to be featured in smartphones and other consumer electronics; however, their ranges are limited, owing to flood illumination. Scanning LiDARs with collimated laser beams have much longer reach but often require bulky scanners that are difficult to integrate.

Recently, there has been intensive research on integrated optical beam scanners capable of high-speed operation over a large FoV with high resolution and low power consumption, which are the key requirements for solid-state LiDARs. Two common architectures are the optical phased array (OPA) and the FPSA. OPAs have been demonstrated on photonic integrated circuit platforms^9–19^ and MEMS platforms^20–22^. They are capable of random-access beam scanning but require precise amplitude and phase control of all the optical antennas in the array, which makes scaling challenging. So far, most OPAs with large pixel number and large FoV are only 1D arrays. Scanning in the orthogonal direction is achieved by wavelength tuning using a widely tunable laser. For example, Chung et al. demonstrated a 1,024-element 1D OPA with 45° FoV in 2018 (ref. ^13^) and Poulton et al. demonstrated an 8,192-element 1D OPA with 100° FoV in 2020 (ref. ^19^). 2D OPAs with large pixel number have been reported on MEMS platforms^21,22^ but their FoV is usually small, owing to the large array pitch to accommodate the MEMS actuators. The performances of some state-of-the-art OPAs are summarized in Extended Data Table 1.

**Extended Data Fig. 1:**
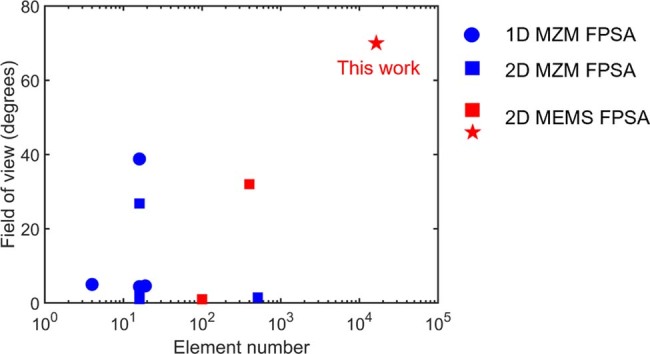
Comparison of integrated FPSAs in the literature and this work. 1D and 2D arrays are indicated by dots and squares, respectively, and this work is indicated by a star.

By contrast, the FPSA uses a camera-like optical system that maps each angle within the FoV to a pixel at the back focal plane of an imaging lens. Instead of integrating a ranging unit at each pixel, the optical switch network in the FPSA allows all pixels to share one (or more) LiDAR ranging unit(s). Each pixel consists of only an optical antenna and a switch, making it possible to integrate a large array on a single chip. Small-scale FPSAs with tens of pixels have been demonstrated using thermally tuned Mach–Zehnder interferometer (MZI) switches^23–29^. However, their integration density is limited by the large footprint of the MZIs and the high power consumption of thermo-optic phase shifters. MEMS-based silicon photonic switches offer many advantages, including small footprint, low loss (nearly zero loss in the OFF state), low power consumption and fast switching time. These advantages have been demonstrated in large-scale optical switches for optical communication networks^30,31^. Proof-of-concept scanners with a small number of pixels (≤400) were recently reported^32,33^. The performances of the reported FPSAs are summarized in Extended Data Table 2 and Extended Data Fig. 1.

In this paper, we experimentally demonstrate a 16,384-pixel FMCW imaging LiDAR with a monolithically integrated 128 × 128-element silicon photonic MEMS FPSA (a 128 × 96 subarray is wire bonded and tested in experiments). With a 5-mm-focal-length compound lens, the system can randomly direct the laser beam to 16,384 distinct directions in a 70° × 70° FoV with a 0.05° divergence angle and a microsecond switching time. To the best of our knowledge, this is the largest integrated 2D FPSA ever reported. 3D imaging is realized by combining the FPSA with FMCW ranging. The FPSA presented here is highly scalable. Megapixel 3D imaging LiDAR is possible by leveraging the Moore’s law-like scaling that has propelled the explosive growth of CMOS imaging sensors over the past few decades.

## FPSA scanner architecture and design

**Fig. 1:**
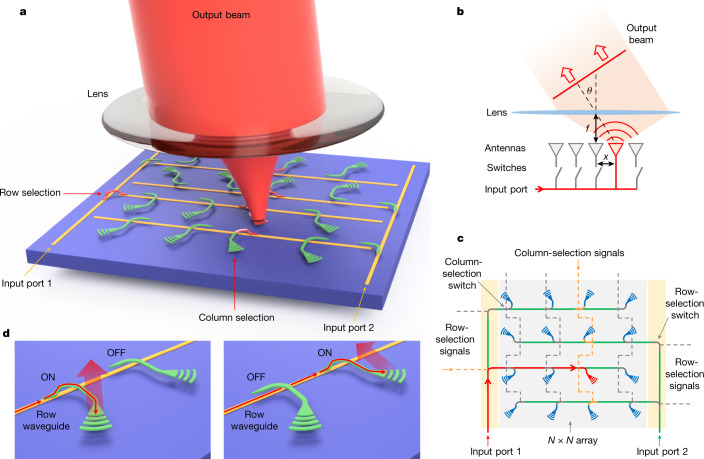
Architecture and working principle of the FPSA. **a**, Perspective-view schematic of the 2D FPSA with the lens and output beam. Light is coupled onto the FPSA chip by means of one of the input ports and then routed to the selected grating antenna by turning on the corresponding row-selection and column-selection switches. The lens converts the emitted light to a collimated beam. **b**, Schematic of a 1D FPSA beam scanner demonstrating the working principle. **c**, Top-view schematic of the 2D FPSA design. **d**, Schematics of the MEMS optical switches and grating antennas in the ON and OFF states. In the ON state, the tip of the polysilicon coupler waveguide (green) is pulled down close to the bus waveguides (yellow) to couple light to the grating antenna.

The schematic of the FPSA is shown in Fig. 1a. The working principle of the beam scanner is shown in Fig. 1b using a 1D FPSA as an example. An array of optical antennas is placed on the back focal plane of a convex lens with a focal length of *f* and each antenna is connected to the input light source by means of an optical switch. When one of the optical switches is turned on, the input light is routed to the corresponding antenna. The emitted light from the antenna is then converted to a collimated beam by the lens. The angle between the output beam and the lens optical axis is *θ* = tan^−1^(−*x*/*f*), in which *x* is the coordinate of the emitting antenna relative to the optical axis. By turning on antennas at different locations, the output beam can be steered to the corresponding angles. The FoV is 2tan^−1^(*L*/2*f*), in which *L* is the overall size of the array. The divergence angle of the output beam can be estimated by *l*/*f*, in which *l* is the size of an individual grating antenna. The angular resolution can be estimated by *p*/*f*, in which *p* is the array pitch.

The architecture of the 2D FPSA is shown in Fig. 1c using a 4 × 4 array as an example. Each optical antenna (grating) is connected to a row waveguide by means of a MEMS optical switch (hereafter called a column-selection switch) and each row waveguide is connected to one of the two input waveguides by means of a MEMS optical switch (hereafter called a row-selection switch). To reduce the total number of control signals from *N*^2^ to 2*N* in an *N* × *N* FPSA, we used a row–column addressing scheme: all switches in the same column are electrically connected, whereas the row-selection switch is individually addressed. By turning on one row-selection switch and one column-selection switch, light from one of the two input ports is routed to the selected grating antenna and emitted to the prescribed angle in free space.

We used a MEMS optical switch design similar to those reported in refs. ^30,31^, in which the tip of a polysilicon coupler is physically moved by an electrostatic MEMS actuator to control the light path, as shown in Fig. 1d. The row-selection switch has two actuators coupling light between the input and row waveguides, whereas the column-selection switch has only one actuator, as the grating is directly connected to the polysilicon waveguide.

**Fig. 2:**
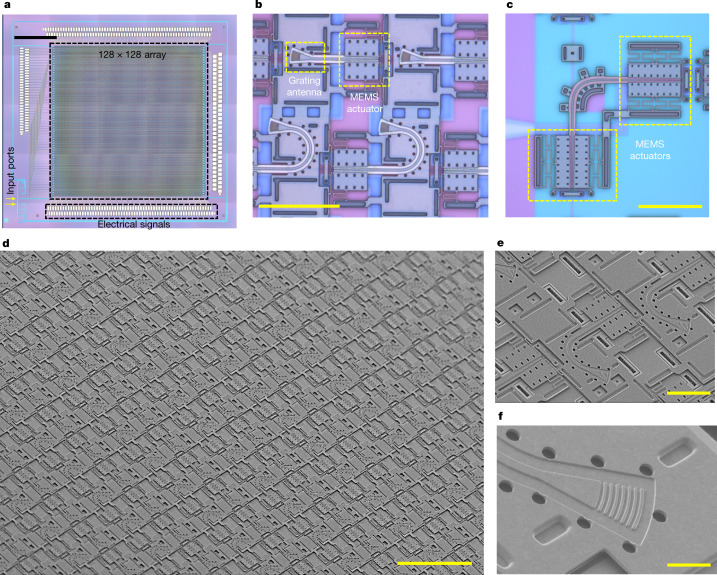
Microscopic images of the fabricated FPSA device. **a**–**c**, Microscopic images showing the FPSA chip (**a**), the grating antennas with column-selection switches (**b**) and the row-selection switch (**c**). **d**–**f**, Scanning electron micrographs of the FPSA chip (**d**), the column-selection switches (**e**) and the grating antennas (**f**). Scale bars: **a**, 2 mm; **b** and **c**, 40 μm; **d**, 100 μm; **e**, 20 μm; **f**, 4 μm.

**Extended Data Fig. 2:**
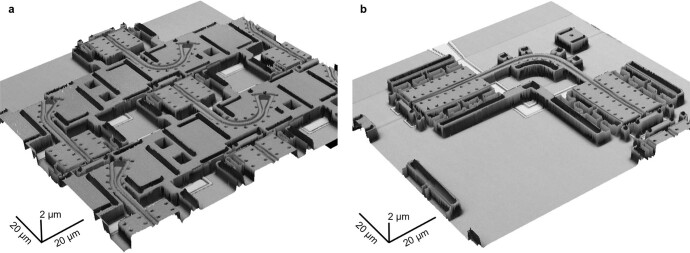
Confocal microscopic images of the fabricated FPSA device. **a**, Confocal microscopic image showing the grating antennas with column-selection switches. **b**, Confocal microscopic image showing the row-selection switch.

**Extended Data Fig. 3:**
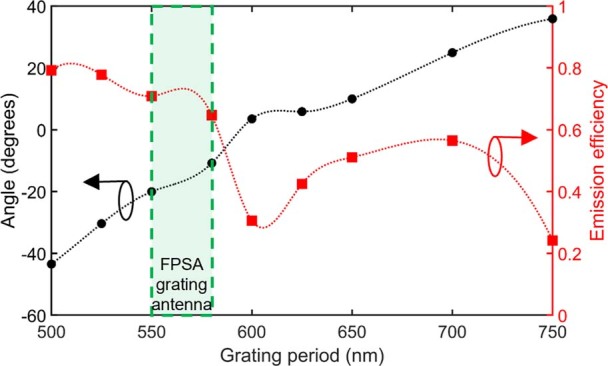
FDTD simulation results of the grating antenna. The emission angle and efficiency as a function of the grating period are simulated for a focusing-curved grating antenna of 10 μm × 5 μm in size. The green-shaded region shows the grating periods selected for the FPSA grating antenna design.

We designed a 128 × 128-element FPSA with a 55-μm pitch in both directions, thus the overall array size is 7 × 7 mm^2^. The overall chip including the routing waveguides, input/output couplers and bonding pads is 10 × 11 mm^2^ in size. The device is fabricated on a silicon-on-insulator (SOI) wafer with a 220-nm-thick device layer. Figure 2a–c shows the optical microscope images of the entire chip, unit cells comprising grating antennas and column-selection switches, and the row-selection switches, respectively. The scanning electron micrographs of the array are shown in Fig. 2d, with the close-up views of the unit cell and the grating antenna in Fig. 2e, f, respectively. Confocal microscopic images of the device are shown in Extended Data Fig. 2. The waveguides are defined on the device layer of the SOI wafer by a partial etch. The grating antennas are patterned on the polysilicon layer with a focusing-curved shape of around 10 × 5 μm^2^ in size. The orientation and grating period of each antenna are individually tailored so that the output beam points towards the centre of the lens to increase the light-collection efficiency and reduce the aberration effects of the lens. The emission efficiencies of all the gratings are kept higher than 65%. The details of the grating antenna design are described in Methods and Extended Data Fig. 3. The device lens (Thorlabs MVL5M23, *f* = 5 mm, F/2.8) maps each grating antenna to a distinctive far-field angle, resulting in a 70° × 70° FoV, 0.6° resolution and 0.05° divergence angle.

## Device characterization

The fabricated FPSA device is wire bonded to a ceramic pin grid array (PGA) and controlled by a field-programmable gate array (FPGA) and a driver circuit board (details are described in Methods). Owing to the limited PGA pin count, only three-quarters of the row-addressing signals and all column-addressing signals are bonded, skipping every fourth row of the FPSA.

**Fig. 3:**
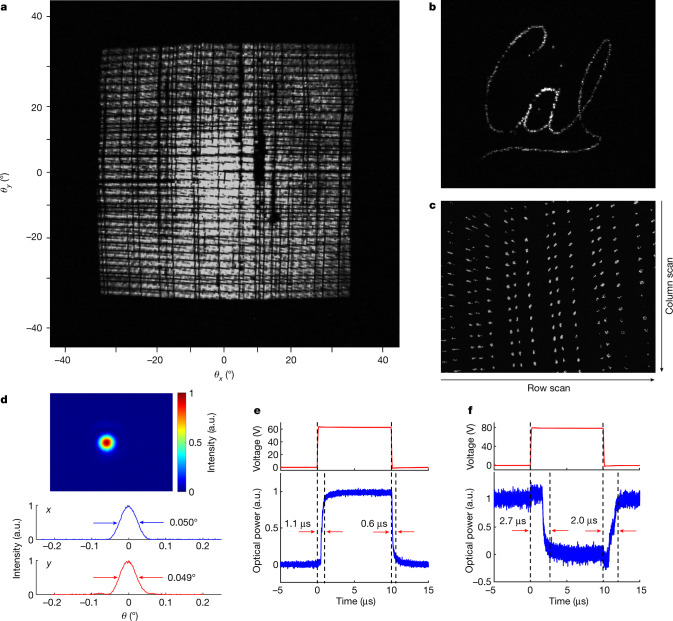
Characterization of the FPSA beam scanner. **a**, Beam-steering pattern projected on a paper screen showing a 70° × 70° FoV. **b**, A scanned ‘Cal’ logo pattern with 475 distinct output beam directions projected on a paper screen. **c**, Zoomed-in beam-steering pattern captured at the focal plane of a Fourier lens. **d**, Beam profile measured at 0.71 m away from the FPSA beam scanner. **e**, **f**, Dynamic responses of the row-selection switch (average of 22 measurements of optical power measured at the drop port) (**e**) and the column-selection switch (average of 32 measurements of optical power measured at the through port) (**f**) in the FPSA. The red curves show the applied voltage waveform and the blue curves show the measured optical power.

**Extended Data Fig. 4:**
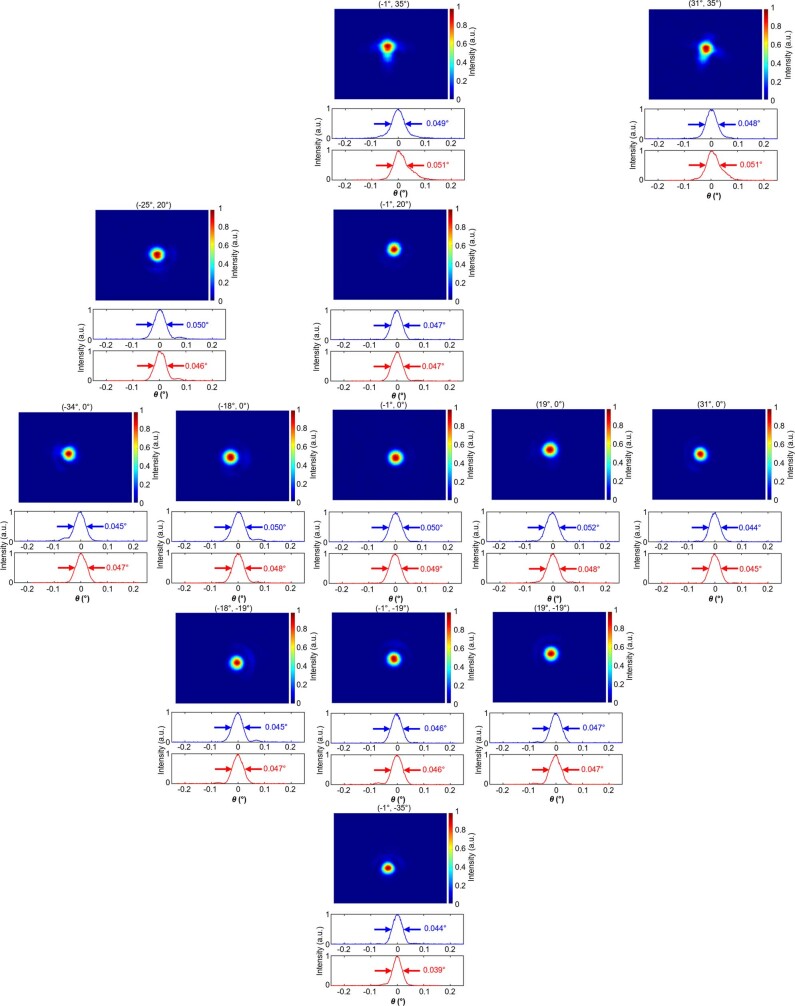
Measured beam profiles of 13 pixels across the FPSA. The beam profiles are measured at 0.71 m away from the FPSA beam scanner.

**Extended Data Fig. 5:**
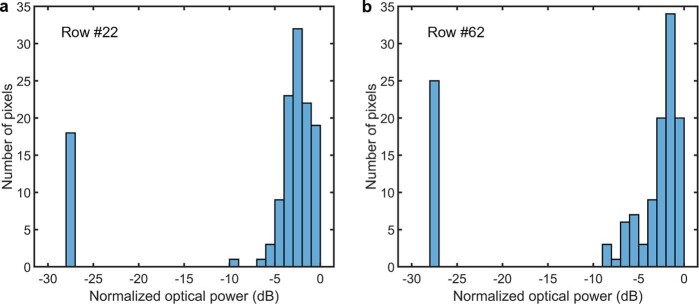
Histograms of measured free-space output power of the FPSA beam scanner. The output power is measured from 128 pixels in row 22 (**a**) and 128 pixels in row 62 (**b**). The bars at −27 dB represent pixels with no output power measured.

**Extended Data Fig. 6:**
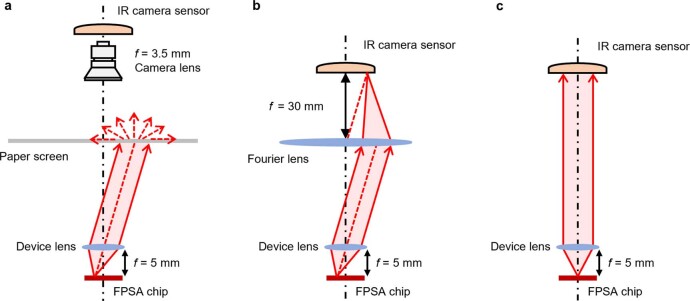
Schematics of the optical characterization setups. **a**, Schematic of the setup for capturing the beam-steering pattern in the 70° × 70° FoV. **b**, Schematic of the setup for capturing the zoomed-in beam-steering pattern. **c**, Schematic of the setup for measuring the beam profiles.

To demonstrate optical beam steering, the electrically addressed 128 × 96 grating antennas are turned on one at a time. The captured images of the output beam projection onto a paper screen are overlapped and shown in Fig. 3a. This clearly demonstrates that the fabricated FPSA is able to steer the output beam across the 70° × 70° FoV as designed, albeit with a small number of dark spots caused by minor defects on the device. To demonstrate the random-access beam-steering capability, we program the FPSA to sequentially turn on 475 selected grating antennas to display a ‘Cal’ logo, as shown in Fig. 3b. Figure 3c shows an enlarged far-field pattern with the output beam raster scanned at 100 kHz and captured by a single frame of the infrared camera running at 33 frames per second. We also characterized the beam quality by measuring the far-field beam profile with an infrared camera at 0.71 m distance away from the lens. Figure 3d shows that the measured full width at half maximum beam divergence is 0.050° × 0.049°, which matches well with the diffraction limit of the lens aperture. Measured beam profiles of more pixels are shown in Extended Data Fig. 4 and output-power-variation measurement results are shown in Extended Data Fig. 5. Details of the optical characterization setups are described in Methods and Extended Data Fig. 6.

The temporal responses of the row-selection and column-selection switches are characterized by measuring the optical power at the switch output ports while applying a square-wave voltage signal, as shown in Fig. 3e, f, respectively. The on and off response times of the row-selection switch are 1.1 μs and 0.6 μs, respectively. The response times of the column-selection switch are slightly longer (2.7 μs and 2.0 μs for on and off switching, respectively), owing to the more compact design of the MEMS actuator. The results indicate that the device can be operated at a sub-MHz frequency for beam steering, which is suitable for scanning LiDARs.

## 3D imaging

**Fig. 4:**
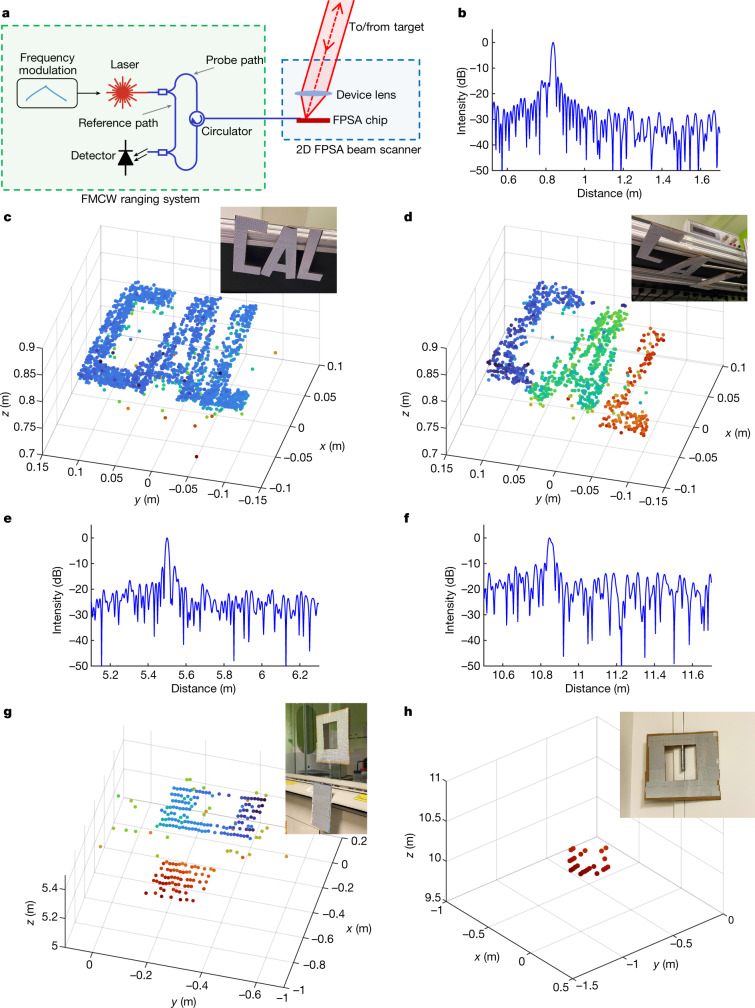
3D imaging results. **a**, Schematic of the FMCW LiDAR with the FPSA beam scanner. **b**, Representative FMCW LiDAR spectrum with a target at 0.84 m. **c**, **d**, Point clouds and camera images of the targets composed of three letters in the same plane (**c**) and in different planes (**d**) at about 0.8 m. **e**, **f**, Representative FMCW LiDAR spectra with targets at 5.5 m (**e**) and 10.8 m (**f**). **g**, **h**, Point clouds and camera images of targets at about 5.2 m (**g**) and 10 m (**h**). The point clouds are colour-coded by the *z*-coordinate values.

**Extended Data Fig. 7:**
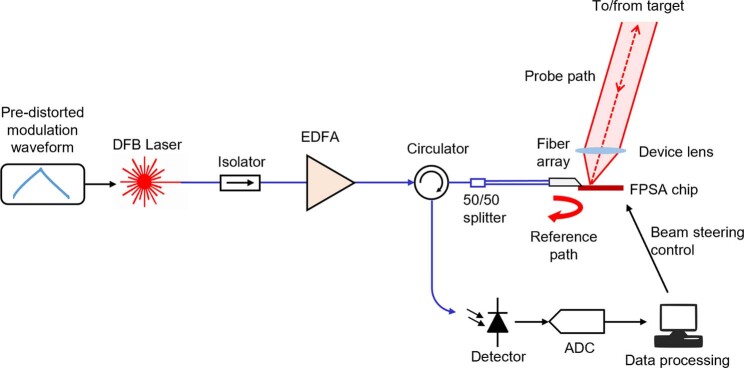
Optical and electrical control setup of the FMCW LiDAR with the FPSA beam scanner for 3D imaging. The LiDAR is operated in a monostatic configuration, in which the same grating antenna on the FPSA is used to transmit the FMCW light and receive the returned signal from the target.

An imaging LiDAR is constructed by combining the FPSA with a frequency-modulated laser and a coherent receiver. A schematic of the system is shown in Fig. 4a, with further details described in Methods and Extended Data Fig. 7. The components of the FMCW ranging system are off-chip in this demonstration; however, they could also be integrated on-chip, as demonstrated by Bhargava et al.^34^. Linear frequency chirps with an excursion of 8.6 GHz and a ramp time of 80 μs are generated by directly modulating a 1,550 nm-wavelength distributed feedback (DFB) laser with a pre-distorted waveform obtained from an iterative learning method^35^. The returned light from the target is mixed with a reference light at the photodetector. A Fourier transform then extracts the beat frequency that is proportional to the target distance. We use a monostatic configuration in which the same grating antenna on the FPSA is used to transmit the FMCW light and receive the returned signal from the target.

To demonstrate 3D imaging, we used a 25-mm-focal-length F/1.4 lens (Navitar SWIR-25) with the FPSA, achieving a 16° × 16° FoV and a 0.13° addressing resolution. Reflective targets made of materials similar to those used for traffic signs are placed at distances of roughly 0.8 m, 5 m and 10 m from the imaging lens. The output beam is scanned sequentially in the FoV. The LiDAR ranging resolution is 1.7 cm and the output power at the device lens is about 1 mW for the 0.8-m measurements and 2 mW for the 5-m and 10-m measurements. Example beat signal spectra of the FMCW LiDAR measurements are shown in Fig. 4b, e, f and the measured point clouds are shown in Figs. 4c, d, g, h. Good 3D image fidelity is achieved with lateral resolution matched to that of the FPSA and distance resolution agreeing well with the frequency excursion.

## Scalability of imaging LiDAR

To further increase the resolution, we can increase the chip size and/or shrink the footprint of the pixel. The footprint of the current pixel (55 × 55 μm^2^) can be reduced by optimizing the design of the MEMS actuators and the switch couplers. It is feasible to shrink the pixel to 10 × 10 μm^2^ for megapixel LiDAR with a 1 × 1-cm^2^ FPSA, which will improve the angular resolution to 0.11° with an *f* = 5 mm lens or 0.02° with an *f* = 25 mm lens. For such high-resolution FPSA, it is important to minimize the switch loss, as there are a large number of switches (1,000) along each row. A salient feature of our FPSA is that the MEMS-actuated switches have nearly zero loss in the OFF state (the only loss is waveguide propagation loss), unlike the MZI-based thermo-optic switches. This makes it possible to keep the optical insertion loss low for high-density FPSAs. The FPSA is fabricated using standard semiconductor processes and can be mass-produced at commercial CMOS foundries.

One unique advantage of the FPSA LiDAR is its flexibility. The FoV and angular resolution can be easily adjusted by selecting imaging lenses with different focal lengths, taking advantage of the large library of well-optimized camera lenses designed for a wide range of focal lengths and image sensor sizes. For example, compact mobile phone camera lenses are suitable for small FPSA chips aiming at a small footprint and large FoV, whereas lenses designed for professional cameras are suitable for large FPSA chips aiming at low divergence and high angular resolution. Full 180° or even larger FoV can also be achieved with fisheye lenses.

## Conclusions and discussions

We have presented the performance of a large-scale (16,384-pixel) imaging LiDAR using a 128 × 128 silicon photonic FPSA, in which a 128 × 96 subarray is wire bonded and tested in experiments. The grating antenna in each pixel is digitally controlled by an integrated MEMS optical switch within an area of 55 × 55 μm^2^. Random-access beam steering with a FoV of 70° × 70°, an addressing resolution of 0.6° in both directions, a beam divergence of 0.05° and a sub-MHz operation speed is achieved with a 5-mm-focal-length imaging lens. 3D imaging with 1.7-cm range resolution has also been demonstrated. The angular resolution of the current system can be further increased by optimizing the optical design and fabrication technology. In addition to 3D sensing applications, the FPSA can also be used in other applications that require optical beam steering, such as free-space optical communication^36^ and trapped-ion quantum computation^37^.

## Methods

### Comparison of FPSAs and OPAs

Extended Data Table 1 compares the performance of state-of-the-art OPAs reported in the literature. Extended Data Table 2 and Extended Data Fig. 1 compare the performance of the FPSAs reported here with other 1D and 2D FPSAs reported in the literature. Note that, although some of the 1D FPSAs and OPAs use wavelength tuning to steer the beam in the orthogonal direction, here we only summarize their beam-steering performances in the FPSA or OPA direction.

### Device fabrication and characterization

The FPSA device fabrication process starts with a standard silicon photonics process on SOI, followed by the deposition and patterning of an extra polysilicon layer for MEMS actuators, polysilicon coupler waveguides and grating antennas, similar to that described in ref. ^30^. Extended Data Figure 2 shows confocal microscopic images of the fabricated FPSA device.

The far-field beam profiles of 13 pixels in addition to the one shown in Fig. 3d are measured with an infrared camera at a distance of 0.71 m away from the lens (the same condition as for Fig. 3d) and the beam profiles and cross sections are shown in Extended Data Fig. 4. The results show that the beam divergence angles are consistent throughout the array, with an average divergence of 0.048° × 0.047° and a standard deviation of 0.0026° × 0.0029°.

The free-space output power from 256 pixels (128 pixels in row 22 and 128 pixels in row 62) of the FPSA beam scanner after the device lens is measured by an optical power meter and histograms of the normalized optical power are shown in Extended Data Fig. 5. The data show that 105 of 128 pixels (82%) in row 22 and 86 of 128 pixels (67%) in row 62 have an output-power variation within 5 dB. The output-power variation is partly caused by the variation of optical losses; that is, light emitted from grating antennas at different locations experience different waveguide propagation losses, grating emission losses and lens collection losses, which will be further discussed in the ‘Optical efficiency’ section. Fabrication imperfections also contribute to the output-power variation. Reducing optical losses and improving the fabrication process will help improve the power uniformity. The pixels with no measured output power (corresponding to dark spots in Fig. 3a) are attributable to damaged MEMS actuators or electrical connections caused by fabrication and handling imperfections, and we expect that the yield can be largely improved with professional nanofabrication foundries.

### Grating antenna design and simulation

The grating antennas are patterned on the 350-nm-thick polysilicon layer with a 250-nm partial etch. Each grating antenna has seven grooves with a constant width of 290 nm. We customize the grating periods according to the grating locations in the array so that the output light is directed towards the centre of the lens aperture. This will increase the lens collection efficiency and reduce aberrations. Finite-difference time-domain (FDTD) simulation results of the grating emission angle and efficiency as a function of the grating period are shown in Extended Data Fig. 3. The simulations show that the grating emission efficiency will notably decrease when the output is close to 0° (vertical direction). On the other hand, the grating pattern width will approach the minimum feature size of our lithography (ASML DUV Stepper Model 5500/300) when the output angle is smaller than −20°. Considering these trade-offs, we set the output directions of the grating antennas to be from −10° to −20° from the vertical direction, corresponding to grating periods in the range 550–580 nm. This is by no means a fundamental limit for grating antenna design, and a higher emission efficiency can be achieved with an optimized design of grating geometry^38,39^.

### Optical efficiency

The on-chip optical losses of the FPSA reported here mainly come from three sources: (1) waveguide propagation loss (3.8 dB cm^−1^); note that grating antennas at different locations are connected to different waveguide lengths and, thus, have different waveguide propagation losses; (2) row-selection-switch loss (2 dB) and column-selection-switch loss (2.5 dB); (3) grating antenna emission loss (about 1.9 dB, depending on the grating antenna locations, as shown in Extended Data Fig. 3). For example, a grating antenna with a 1-cm waveguide has an on-chip loss of 10.2 dB. By optimizing the parameters in the fabrication process for the same device layout, we can reduce the waveguide propagation loss to 1 dB cm^−1^ and the switch losses to less than 1 dB for both row-selection and column-selection switches, therefore the on-chip loss of a grating antenna with a 1-cm waveguide can be reduced to 4.9 dB. The on-chip optical efficiency can be further improved by optimizing the design of grating antennas and MEMS switch actuators.

In addition to the on-chip losses, the demonstrated LiDAR setup also has two off-chip losses: (1) fibre-to-chip coupling loss (5 dB for each coupling) and (2) loss owing to limited device lens transmittance and collection efficiency of grating antenna emitted light (total about 3 dB). The fibre-to-chip coupling loss can be eliminated if the LiDAR ranging system components are integrated on-chip^34^. The device lens loss can be reduced by applying anti-reflection coating for the operating wavelength, as well as improving the optical design to better match the lens aperture with the grating antenna emitting pattern.

### Packaging and control

The FPSA chip is attached and wire bonded to a 256-pin ceramic PGA. Owing to the in-plane fibre array for optical I/O coupling, some electrical pads on the PGA are blocked by the fibre array, therefore the total number of available pads is smaller than the required control signals (256 + ground). Instead of missing a contiguous block of the array, every fourth row of the array is skipped for wire bonding, whereas all columns are wire bonded, so a 128 × 96 subarray is tested in experiments. A driver circuit board with two HV583 (128-channel low-voltage serial to high-voltage parallel converter) chips generates the electrical control signals for the FPSA and the output is controlled by an FPGA. The driver circuit board can update the FPSA electrical control signal at a rate of up to 1.25 MHz for random-access beam steering. Light is coupled between the external fibre-based optical setup and the FPSA chip using an off-chip fibre array and on-chip grating couplers.

### Optical characterization methods and setup

The beam-steering patterns in the 70° × 70° FoV shown in Fig. 3a, b are captured by projecting the output beam from the 5-mm-focal-length lens on a sheet of paper as a diffuser screen and imaging the screen pattern using an infrared camera (Xenics Bobcat 320) with a 3.5-mm-focal-length wide-angle lens. The optical setup is shown in Extended Data Fig. 6a.

The zoomed-in far-field beam-steering pattern shown in Fig. 3c is measured by collecting the output beam from the device lens using a 30-mm-focal-length lens as the Fourier lens and capturing the intensity distribution on the focal plane of the Fourier lens using the infrared camera sensor, which is effectively the far-field intensity distribution of the beam scanner. The optical setup is shown in Extended Data Fig. 6b.

The beam profiles shown in Fig. 3d and Extended Data Fig. 4 are captured by the bare infrared camera sensor at a distance of 0.71 m away from the device lens, and the optical setup is shown in Extended Data Fig. 6c.

### FMCW LiDAR 3D imaging setup

The optical and electrical setup for the 3D imaging demonstration is shown in Extended Data Fig. 7. The DFB laser (Optilab DFB-1550) is linearly frequency modulated by a pre-distorted current waveform obtained by the iterative learning pre-distortion process^35^. An erbium-doped fibre amplifier boosts the optical power to compensate for the loss along the optical path. The amplified light passes through a fibre circulator and then splits into two paths by a 50/50 splitter for the two input waveguides (for 5-m and 10-m LiDAR measurements, light is coupled into one of the input waveguides). Light is coupled to the FPSA chip by means of an angled polished fibre array and on-chip grating couplers, and then directed to the target from one of the grating antennas through the imaging lens. The returned light from the target is received by the same grating antenna on the FPSA and coupled back to the fibre array. The fibre array facet and the surface of the grating coupler on the FPSA chip together have about −34 dB of reflection, which is used as the reference path (local oscillator) of the FMCW LiDAR. The reference and probe light passes through the circulator to the photodetector (Thorlabs PDB480C-AC), generating a beat signal sampled by an analogue-to-digital converter (National Instruments PXIe-5114). The data are transferred to a laptop computer and the beat frequency that is proportional to the target distance is extracted by performing a fast Fourier transform. For each beam direction, the distance measurements are repeated ten times and the results are averaged to increase the measurement precision. The FPSA chip is also controlled by the laptop computer by means of the FPGA and the driver circuit board.

## Online content

Any methods, additional references, Nature Research reporting summaries, source data, extended data, supplementary information, acknowledgements, peer review information; details of author contributions and competing interests; and statements of data and code availability are available at 10.1038/s41586-022-04415-8.

## Figures and Tables

**Extended Data Table 1:**
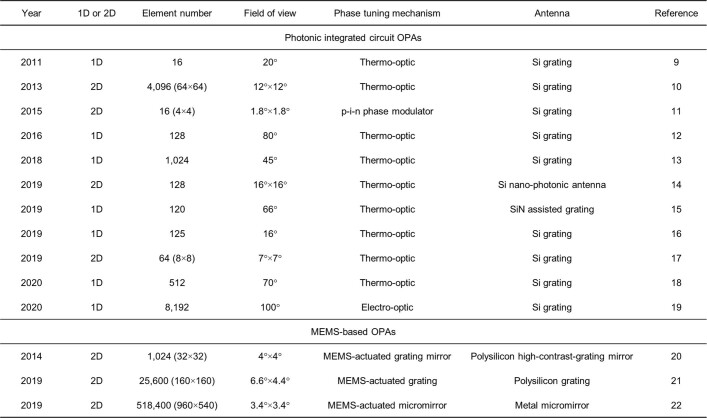
Comparison of OPAs in the literature

**Extended Data Table 2:**
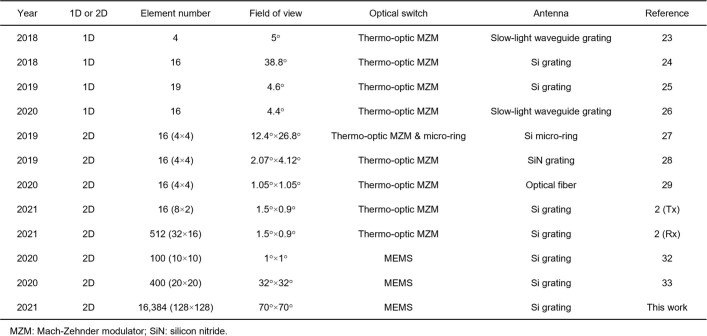
Comparison of integrated FPSAs in the literature and this work

## Data Availability

The data used to produce the plots in this paper and the extended data plots are available in Dryad with the identifier 10.6078/D1HB0C.

## Code availability

The code used to analyse the data and produce the plots are available in Dryad with the identifier 10.6078/D1HB0C.

## References

[1] Schwarz B (2010). Mapping the world in 3D. Nat. Photon..

[2] Rogers C (2021). A universal 3D imaging sensor on a silicon photonics platform. Nature.

[3] Shi J, Guo J, Kagami M, Suni P, Ziemann O (2019). Photonic technologies for autonomous cars: feature introduction. Opt. Express.

[4] Javidi B (2020). Roadmap on 3D integral imaging: sensing, processing, and display. Opt. Express.

[5] Behroozpour B, Sandborn PAM, Wu MC, Boser BE (2017). Lidar system architectures and circuits. IEEE Commun. Mag..

[6] Shin D (2016). Photon-efficient imaging with a single-photon camera. Nat. Commun..

[7] Morimoto K (2020). Megapixel time-gated SPAD image sensor for 2D and 3D imaging applications. Optica.

[8] Kuzmenko K (2020). 3D LIDAR imaging using Ge-on-Si single–photon avalanche diode detectors. Opt. Express.

[9] Doylend JK (2011). Two-dimensional free-space beam steering with an optical phased array on silicon-on-insulator. Opt. Express.

[10] Sun J, Timurdogan E, Yaacobi A, Hosseini ES, Watts MR (2013). Large-scale nanophotonic phased array. Nature.

[11] Aflatouni F, Abiri B, Rekhi A, Hajimiri A (2015). Nanophotonic projection system. Opt. Express.

[12] Hutchison DN (2016). High-resolution aliasing-free optical beam steering. Optica.

[13] Chung S, Abediasl H, Hashemi H (2018). A monolithically integrated large-scale optical phased array in silicon-on-insulator CMOS. IEEE J. Solid-State Circuits.

[14] Fatemi R, Khachaturian A, Hajimiri A (2019). A nonuniform sparse 2-D large-FOV optical phased array with a low-power PWM drive. IEEE J. Solid-State Circuits.

[15] Zhang Y (2019). Sub-wavelength-pitch silicon-photonic optical phased array for large field-of-regard coherent optical beam steering. Opt. Express.

[16] Kim T (2019). A single-chip optical phased array in a wafer-scale silicon photonics/CMOS 3D-integration platform. IEEE J. Solid-State Circuits.

[17] Ashtiani F, Aflatouni F (2019). N × N optical phased array with 2N phase shifters. Opt. Express.

[18] Miller SA (2020). Large-scale optical phased array using a low-power multi-pass silicon photonic platform. Optica.

[19] Poulton, C. V. et al. 8192-element optical phased array with 100° steering range and flip-chip CMOS. In *2020 Conference on Lasers and Electro-Optics (CLEO)* paper JTh4A.3 (Optical Society of America, 2020).

[20] Yoo B (2014). A 32 × 32 optical phased array using polysilicon sub-wavelength high-contrast-grating mirrors. Opt. Express.

[21] Wang Y (2019). 2D broadband beamsteering with large-scale MEMS optical phased array. Optica.

[22] Bartlett, T. A., McDonald, W. C. & Hall, J. N. Adapting Texas Instruments DLP technology to demonstrate a phase spatial light modulator. In *Emerging Digital Micromirror Device Based Systems and Applications XI* paper 109320S (International Society for Optics and Photonics, 2019).

[23] Abe H (2018). Two-dimensional beam-steering device using a doubly periodic Si photonic-crystal waveguide. Opt. Express.

[24] López, J. J. et al. Planar-lens enabled beam steering for chip-scale LIDAR. In *2018 Conference on Lasers and Electro-Optics (CLEO)* paper SM3I.1 (Optical Society of America, 2018).

[25] Inoue D, Ichikawa T, Kawasaki A, Yamashita T (2019). Demonstration of a new optical scanner using silicon photonics integrated circuit. Opt. Express.

[26] Ito H (2020). Wide beam steering by slow-light waveguide gratings and a prism lens. Optica.

[27] Chang, Y. et al. Metalens-enabled low-power solid-state 2D beam steering. In *2019 Conference on Lasers and Electro-Optics (CLEO)* paper SF3N.5 (Optical Society of America, 2019).

[28] Li C, Cao X, Wu K, Li X, Chen J (2019). Lens-based integrated 2D beam-steering device with defocusing approach and broadband pulse operation for Lidar application. Opt. Express.

[29] Cao X, Qiu G, Wu K, Li C, Chen J (2020). Lidar system based on lens assisted integrated beam steering. Opt. Lett..

[30] Seok TJ, Quack N, Han S, Muller RS, Wu MC (2016). Large-scale broadband digital silicon photonic switches with vertical adiabatic couplers. Optica.

[31] Seok TJ, Kwon K, Henriksson J, Luo J, Wu MC (2019). Wafer-scale silicon photonic switches beyond die size limit. Optica.

[32] Cook EH (2020). Polysilicon grating switches for LiDAR. J. Microelectromech. Syst..

[33] Zhang, X., Kwon, K., Henriksson, J., Luo, J. & Wu, M. C. A 20x20 focal plane switch array for optical beam steering. In *2020 Conference on Lasers and Electro-Optics (CLEO)* paper SM1O.3 (Optical Society of America, 2020).

[34] Bhargava, P. et al. Fully integrated coherent LiDAR in 3D-integrated silicon photonics/65nm CMOS. In *2019 Symposium on VLSI Circuits* C262–C263 (IEEE, 2019).

[35] Zhang X, Pouls J, Wu MC (2019). Laser frequency sweep linearization by iterative learning pre-distortion for FMCW LiDAR. Opt. Express.

[36] Kaymak Y (2018). A survey on acquisition, tracking, and pointing mechanisms for mobile free-space optical communications. IEEE Commun. Surv. Tutor..

[37] Kim, J. et al. Enabling trapped ion quantum computing with MEMS technology. In *2017 International Conference on Optical MEMS and Nanophotonics (OMN)* (IEEE, 2017).

[38] Michaels A, Yablonovitch E (2018). Inverse design of near unity efficiency perfectly vertical grating couplers. Opt. Express.

[39] Khajavi S (2021). Compact and highly-efficient broadband surface grating antenna on a silicon platform. Opt. Express.

